# Supplementary material for: Gut microbiome differences among Mexican Americans with and without type 2 diabetes mellitus
Source: PLoS One. 2021 May 13;16(5):e0251245. doi: 10.1371/journal.pone.0251245 (PMC8118452; doi:10.1371/journal.pone.0251245)
Supplement: Attachment — Submitted filename: Response to Reviewers_PLoS One.docx [file pone.0251245.s002.docx]

**Response to Reviewers – Manuscript ID PONE-D-21-02685**

**“**Gut microbiome differences among Mexican Americans with and without type 2 diabetes mellitus**”**

**Editor Comments**

| **#** | **Comments** | **Authors’ Response** | | **Manuscript Changes** |
| --- | --- | --- | --- | --- |
| 1 | Please ensure that your manuscript meets PLOS ONE's style requirements, including those for file naming. | Manuscript has been reviewed to ensure compliance with style requirements. | Please see changes throughout the manuscript. | |
| 2 | In your Methods section, please provide additional information about the participant recruitment method and the demographic details of your participants:  a) the recruitment date range (month and year) | **Page 4, lines 76-77**: This was a cross-sectional study of volunteers from San Antonio, TX and surrounding areas from June 2017 to July 2018. | No additional changes. | |
|  | b) a description of any inclusion/exclusion criteria that were applied to participant recruitment | **Page 4, lines 86-92**: Subjects were eligible for inclusion if they were at least 18 years old and self-identified as Mexican American. Subjects were excluded if they had a history of prior gastrointestinal surgery that potentially altered the anatomy of the gastrointestinal tract. Subjects were also excluded for use of specific drugs. Medication use that warranted exclusion included (1) chronic daily use of any drug meant to alter gastrointestinal secretory or motor function (e.g., prokinetic agents, narcotic analgesics, laxatives, anticholinergics, anti-diarrheals) or (2) any use of antibiotics, gastric-acid suppressing medications, or probiotics in the previous two months. | No additional changes. | |
|  | c) a table of relevant demographic details | **Table 1**. Baseline characteristics. | No additional changes. | |
|  | d) a statement as to whether your sample can be considered representative of a larger population | Please see recommended additions. | **Page 4, lines 81-83:** Due to this recruitment method, the sampled population most likely represents relatively healthy community-dwelling individuals and may not be representative of all T2DM and non-diabetic patient populations. | |
|  | e) a description of how participants were recruited and | **Page 4, lines 79-81**: Subjects were recruited using newspaper advertisements in the San Antonio Express-News, Southside Reporter, and Conexión. Flyers were also placed in the Medical Arts and Research Center (MARC) in the South Texas Medical Center. | No additional changes. | |
|  | f) descriptions of where participants were recruited and where the research took place | **Page 4, lines 83-86**: Those interested in participating called the research team, and a research team member pre-screened subjects using a detailed questionnaire. If participants successfully completed pre-screening, they were scheduled for a research visit at the MARC. | No additional changes. | |
| 3 | Please upload these data to a stable, public repository (such as ArrayExpress, Gene Expression Omnibus (GEO), DNA Data Bank of Japan (DDBJ), NCBI GenBank, NCBI Sequence Read Archive, or EMBL Nucleotide Sequence Database (ENA)). In your revised cover letter, please provide the relevant accession numbers that may be used to access these data. | Thanks for this suggestion. We have now uploaded the data into NCBI SRA and have provided the accession number in the manuscript. | N/A. | |
| 4 | Please amend the manuscript submission data (via Edit Submission) to include author Bertha E. Flores | We have amended the online submission to include co-author Bertha Flores. | N/A. | |
| 5 | Please include captions for your Supporting Information files at the end of your manuscript, and update any in-text citations to match accordingly. | We have now included captions for Supporting Information files at the end of the manuscript. | **Page 18**: Supporting Information Files.  S1 Appendix. Clinical and microbial diversity and abundance data. | |
| 6 | Please remove any funding-related text from the manuscript and let us know how you would like to update your Funding Statement. Currently, your Funding Statement reads as follows:  'This study was funded by the Research to Advance Community Health Center, University of Texas San Antonio ([reach.uthscsa.edu](http://reach.uthscsa.edu/)) awarded to KRR. The funders had no role in study design, data collection and analysis, decision to publish, or preparation of the manuscript.'  b. Please include your amended statements within your cover letter; we will change the online submission form on your behalf. | We have removed funding information from the acknowledgements section of the manuscript. Please keep the funding statement as is:  This study was funded by the Research to Advance Community Health Center, University of Texas San Antonio ([reach.uthscsa.edu](http://reach.uthscsa.edu/)) awarded to KRR. The funders had no role in study design, data collection and analysis, decision to publish, or preparation of the manuscript. | **Page 13, Acknowledgements**: funding source removed. | |
| 7 | The data needs to be presented in a more common fashion. I believe the authors are wrongly using the Wilcoxon as their data from two cohorts can't be paired. As suggested by Reviewer 1, Table 2 need to be represented graphically with actual relative abundances. It is absolutely fine that there were no remarkable difference between groups. But the granular differences need to be shown with even more rigor. I would guess that some taxa (Haemophilus, Pyramidobacter) were extremely low abundant, and therefore may be irrelevant to present. | Agreed. Please see our responses to Review 1 regarding presentation of data as relative abundances rather than counts. Regarding comparisons, we used the Wilcoxon rank sum test (rather than the Wilcoxon signed rank test) because our data were unmatched and non-parametric. Since some taxa were in very low abundance, we have chosen to remove these from presentation. | Please see changes outlined under Review 1 below. | |

**Reviewer #1**

| **#** | **Comments** | **Authors’ Response** | | **Manuscript Changes** |
| --- | --- | --- | --- | --- |
| 1 | The microbiome data should be reported as relative abundance, not raw counts. | Agreed. We have changed our reporting of the most common phyla, families, and statistically different OTUs as relative abundance rather than counts. | **Page 8, lines 154-162:** Though not reaching statistical significance, the T2DM group had a higher percent abundance of Firmicutes (36.5% vs. 30.0%, p=0.131), Proteobacteria (6.4% vs. 5.7%, p=0.485), and Verrucomicrobia (5.3% vs. 2.5%, p=0.062) and a lower abundance of Bacteroidetes (49.2% vs. 59.2%, p=0.131) compared to the non-T2DM group (Figure 2). At the family level, the largest numeric differences between the T2DM and non-T2DM groups were with the Bacteroidaceae (28.8% vs. 21.6%, p=0.399) and Prevotellaceae (13.1% vs. 30.2%, p=0.172) though neither reached statistical significance (Figure 3). There was a significant difference in the relative abundance of 16 OTUs between groups. Figure 4 displays the percent abundance of the eight most abundance of these OTUs; p<0.001 for all comparisons between T2DM and non-T2DM groups.  **Figure 2.** Percent relative abundance of bacterial phyla by diabetes status.  **Figure 3.** Percent relative abundance of bacterial families by diabetes status.  **Figure 4.** Percent relative abundance of the most abundant significantly different OTUs. | |
| 2 | Table 2 is difficult to read, but this would probably be addressed by reporting values as relative abundance instead of counts. Also consider reporting the distributions graphically (as in Fig1) rather than as a table. | Agreed. We have included two new figures documenting the mean percent relative abundance between groups |  |  |
| 3 | Table 2 mentions "normalized counts" but there is no description of how this normalization was performed in the Methods section. | We have removed our reporting of normalized counts and changed all reporting to proportional abundance. |  |  |
| 4 | Will the authors make the microbiome data publicly available upon publication? It is common practice to upload all raw sequencing data the NCBI SRA upon publication and cite the SRA Study Accession number in the manuscript. | Thanks for this suggestion. We have now uploaded the data into NCBI SRA and have provided the accession number in the manuscript: BioProject ID PRJNA719138. | **Page 6, lines 124-125**: Raw sequences and metadata have been deposited into the NCBI Sequence Read Archive (BioProject ID PRJNA719138). | |
